# Maslach Burnout Inventory versus Boyko’s Burnout Inventory: a comparative study and methodology

**DOI:** 10.1007/s44192-024-00097-y

**Published:** 2024-09-24

**Authors:** Marina B. Kotova, Vadim N. Kolesnikov, Marina V. Kiseleva, Anton R. Kiselev, Oxana M. Drapkina

**Affiliations:** 1 National Medical Research Center for Therapy and Preventive Medicine, Moscow, 101990 Russia; 2 Petrozavodsk State University, Petrozavodsk, 185910 Republic of Karelia Russia; 3 Science and Innovations LLC, Saratov, 410004 Russia

**Keywords:** Burnout, Maslach Burnout Inventory, Boyko’s Burnout Inventory

## Abstract

**Background:**

In Russia, the following two questionnaires are mainly used to study the burnout syndrome: Maslach Burnout Inventory (MBI) and Boyko’s Burnout Inventory (BBI). Despite the fact that the questionnaires are based on different theoretical models and composition of the scales, they evaluate essentially the same construct. A few published sources provide data on correlations between the results of measuring burnout using these methods. However, the presence of a correlation does not imply the comparability of the methods. The goal of our study was to compare the results of MBI and BBI as well as to develop a methodology for reciprocal recalculation of their burnout estimates.

**Methods:**

MBI and BBI scales were employed to diagnose the burnout syndrome. Our study included 117 men aged 41–44 years. The total scores obtained by the two methods, as well as the subscale scores, were compared using the correlation analysis, the cross-comparative analysis, and the Bland–Altman plot method, while the associations between the results were estimated with odds ratio (OR) and 95% confidence interval (CI).

**Results:**

The total scores (integral indicators based on summing the scores of all subscales and taking into account differences in the weights of scale scores) demonstrated a high similarity in measuring the severity of the burnout. All three dimensions of burnout sensu MBI correlated with the total BBI score.

**Conclusion:**

The comparison (MBI vs. BBI) demonstrated the consistency of the results, which implied the possibility of comparing data yielded by the studies based on the two questionnaires (MBI and BBI).

## Introduction

Burnout is a psychological phenomenon. Its investigation started in 1970s [1–3] and is still in progress. It was suggested that the main cause of the burnout syndrome development is a chronic stress associated with professional activity and generated by problems arising from the interaction with other people. Burnout syndrome is included in the International Classification of Diseases (ICD-10) as Z73 (“S*tress associated with difficulties in maintaining a normal lifestyle*”). In 1976, Christina Maslach proposed a burnout syndrome model that included three main components: emotional exhaustion, depersonalization, and reduction in personal accomplishment [3]. The first and most commonly used generally accepted method for assessing the severity of burnout components was the Maslach Burnout Inventory (MBI), developed by Christina Maslach and Susan E. Jackson [4]. The MBI has undergone several subsequent modifications [5] and is widely used as the gold standard [6] in numerous studies related to the investigation of occupational health and occupational wellness [7–9]. A special group is formed by the studies aimed at assessing the psychometric qualities of the questionnaire in various categories of subjects [10–12].

Along with the adapted Russian version of the MBI [13], Boyko’s Burnout Inventory (BBI) published in 1996 [14] is widely used by Russian scientists. As of May 23, 2023, this questionnaire was cited as a method for assessing burnout in over 10 thousand publications indexed in the Russian scientific electronic library (eLibrary.ru) and was also quite often used in English-language publications of authors from Russia and some other countries (e.g., [15–18]). The BBI was adapted and validated for Bulgaria as well [19–21]. Unlike Christina Maslach, Viktor V. Boyko used the term ‘emotional burnout’ and identified three phases in the formation of the burnout syndrome, corresponding to the phases of stress development sensu Hans Selye: alarm, resistance, and exhaustion [14]. At each phase, a specific set of emotional burnout symptoms is formed. In fact, the concepts of emotional burnout sensu Boyko and burnout sensu Maslach are essentially identical.

It seems logical that many authors were interested in comparing the results of burnout assessment based on MBI and BBI, and conducted on the same sample of study subjects [22–24]. However, single attempts to compare the two questionnaires do not allow recalculation of the results of studies performed using the BBI for comparison with the data obtained by authors using the MBI. Nonetheless, the development of such recalculation technique would create the possibility of unifying the results yielded by the two questionnaires.

The goal of our study was to compare the results of MBI and BBI, and develop a methodology for mutual recalculation of their burnout estimates.

## Materials and methods

### Study subjects

In 1984, to carry out prospective monitoring of the cardiovascular risk factor dynamics, a representative sample from the general population of boys (Moscow school students) born in 1976–1979 was formed. Over 32 years of prospective observation, seven examinations were conducted. After 32 years of this study (medical office visit #7), 117 men aged 41–44 years (with a mean age of 42.9 years) completed the BBI and MBI questionnaires. All included subjects signed the written informed consent.

### Institutional Review Board Statement

This study was carried out in accordance with the Declaration of Helsinki guidelines. Study protocol was approved by the Research Ethics Committee of the National Medical Research Center for Therapy and Preventive Medicine (Protocol No. 01-08/20 of February 04, 2020).

### Maslach Burnout Inventory

In our study, we employed the Russian version of MBI: Maslach Burnout Inventory-Human Services Survey (MBI-HSS), 22 items, adapted by N.E. Vodopyanova in the early 2000s [13]. When adapted for use among Russian-speaking respondents, the original structure of the MBI (22 statements) was fully preserved.

The MBI questionnaire includes three scales: the 9-item emotional exhaustion (EE) scale, the 5-item depersonalization (DP) scale and 8-item personal accomplishment (PA) scale. Applicability (i.e., frequency of occurrence) of statements describing burnout symptoms is assessed by the respondent on a 7-point Likert-type scale (0 = never, 1 = a few times a year or less, 2 = once a month or less, 3 = a few times a month, 4 = once a week, 5 = a few times a week, 6 = every day) [4]. The results of the MBI Russian version [13] were interpreted in full accordance with the original English version [4].

Initially, the interpretation of the questionnaire did not involve the procedure for calculating the entire burnout: merely scale scores were calculated for the three components of the burnout syndrome [4]. For the Russian version of MBI, the researchers at the Bekhterev Institute (St. Petersburg) proposed a formula for calculating the so-called systemic index of burnout syndrome (SIBS) [25]. The formula for determining the SIBS value (*ρ*) is as follows:

$$p = \sqrt {\frac{{(EE_{x} /54)^{2} + (DP_{x} /30)^{2} + (1 - PA_{x} /48)^{2} }}{3}} ,$$

where *EE*_*x*_ is the respondent’s score on the EE scale (54 is the maximum value on this scale); *DP*_*x*_ is the respondent’s score on the DP scale (with the maximum score of 30); *PA*_*x*_ is the respondent’s score on the PA scale (with its maximum possible value of 48).

The *ρ* value ranges from 0 (no burnout whatsoever) to 1 (the most pronounced burnout).

### Boyko’s Burnout Inventory (BBI)

BBI includes 84 statements. An English translation of Boyko’s Inventory and the methodology of its processing is available in our previous publication (see Appendix in [16]).

Based on the BBI results, three phases are distinguished: alarm, resistance, and exhaustion [14]. The first phase (alarm) is characterized by experiencing psychotraumatic events, the emergence of a feeling of dissatisfaction with oneself, a feeling of being caged, along with anxiety and depression. The second phase (resistance) includes inadequate selective emotional response, emotional and ethical disorientation, expansion of the economy of emotions, and reduction of professional duties. The third phase (exhaustion) comprises emotional deficit, emotional avoidance, depersonalization, and psychosomatic and psychovegetative disorders [26].

According to Boyko, the first and second phases (alarm and resistance) are formed virtually simultaneously, while the sequence of formation of the third phase (exhaustion) is not specified. We hypothesize that the exhaustion is formed later as a consequence of the formation of the alarm and resistance phases.

In BBI, 84 items (statements) form 12 scales (four for each burnout phase). In BBI, 84 items form 12 scales (four for each burnout phase). The subject must agree with each statement (response option ‘Yes’) or disagree with it (response option ‘No’). As a result of processing the responses on the basis of the scale scores, the leading symptoms are identified and the degree of formation of each of the three phases is determined. The procedure for determining the stage (severity) of the burnout syndrome for a study subject, based on the results of diagnostics, was not described by the author. Items of the questionnaire scales have different weights associated with the contribution of a particular manifestation of the syndrome to its formation, and these differences are taken into account when calculating the final score on each scale. The book by Viktor V. Boyko [14] did not provide information about the psychometric characteristics of the questionnaire. Normative indicators of severity are similar for all scales and are clearly based on the number of items in the particular scale.

### Statistical data processing

The assessment of the normality of distribution for each quantitative indicator was carried out on the basis of the Kolmogorov–Smirnov criterion. The internal consistency of the questionnaire scales was assessed based on the calculation of Cronbach’s alpha (values of 0.71 and above were considered sufficient) and Pearson correlation coefficient.

Bland–Altman plot method, cross-comparative analysis, and calculation of odds ratio (OR) with 95% confidence interval (95% CI) were used to compare MBI and BBI results.

Confirmatory factor analysis (the extraction method: principal component analysis; the rotation method: varimax rotation with Kaiser normalization) was performed to examine the factorial validity of the questionnaires (analysis of the correspondence of the theoretical model to the factorial solution for questionnaire items).

All statistical procedures were performed in SPSS (PASW) version 22 (IBM Corp. USA). The critical level of statistical significance was assumed at 0.01.

## Results

### Psychometric characteristics of Maslach Burnout Inventory

The values of test scores of all MBI scales corresponded to a normal distribution. The psychometric characteristics of the MBI scales are presented in Table 1. The internal consistency of the MBI scales (based on Cronbach’s α) was sufficient. The correlation coefficients between the three scales ranged from 0.37 to 0.62. EE and DP correlated the most. The correlation between EE and PA was negative and also quite strong: r = − 0.37, p < 0.001. Factor analysis combined all three scales into a single factor (Table 2), for which the explained proportion of variance was 65.88%.

**Table 1:** Descriptive statistics and correlation coefficients for MBI scales

Descriptive statistics	EE	DP	PA	SIBS
M	18.57	8.11	31.32	0.34
SD	8.49	4.96	6.30	0.12
Z	0.586	0.734	0.857	0.869
Asymp. Sig. (2-tailed)	0.882	0.654	0.455	0.437
Internal consistency (Cronbach’s alpha)	0.844	0.773	0.713	
*Correlation coefficients for MBI scales*				
EE		0.624^*^	− 0.369^*^	0.851^*^
DP			− 0.454^*^	0.831^*^
PA				− 0.725^*^

*M* mean, *SD* standard deviation, *Z* Z-score in Kolmogorov–Smirnov test, *Asymp. Sig. (2-tailed)* asymptotic significance (two-tailed) in Kolmogorov–Smirnov test, *EE* emotional exhaustion, *DP* depersonalization, *PA* personal accomplishment, *SIBS* systemic index of burnout syndrome

*p < 0.01

**Table 2:** Factor matrix of MBI scales

MBI scales	Component
EE	0.837
DP	0.866
PA	− 0.722
PVE, %	65.88

Extraction method: principal component analysis; 1 component extracted. *PVE* proportion (%) of variance explained by the factor. *EE* emotional exhaustion, *DP* depersonalization, *PA* personal accomplishment

As a result of the confirmatory factor analysis of 22 MBI items, three factors were identified (Table 3).

**Table 3:** Factor matrix of MBI items

MBI items	Factors		
	1 (25.51%)	2 (14.58%)	3 (8.52%)
Item 20 EE	0.753	− 0.367	
Item 8 EE	0.737		
Item 3 EE	0.735		
Item 1 EE	0.727		
Item 11 DP	0.679		
Item 2 EE	0.665		− 0.301
Item 22 DP	0.622		
Item 16 EE	0.590		− 0.441
Item 13 EE	0.579	− 0.391	
Item 5 DP	0.550	− 0.360	
Item 9 PA		0.696	
Item 12 PA		0.626	
Item 19 PA		0.622	
Item 15 DP	0.504	− 0.556	
Item 10 DP	0.474	− 0.519	
Item 14 EE	0.459	0.490	
Item 18 PA	− 0.414	0.447	0.312
Item 4 PA		0.359	
Item 7 PA			0.678
Item 17 PA		0.426	0.610
Item 6 EE	0.320		− 0.420
Item 21 PA	− 0.313		0.384

The numbers correspond to the ordinal numbers of the questionnaire items, the letter codes indicate the scales: *EE* emotional exhaustion, *DP* depersonalization, *PA* reduction of personal accomplishment; factor loadings of 0.3 and higher are presented

### Psychometric characteristics of Boyko’s Burnout Inventory

Score values of all BBI scales corresponded to a normal distribution. The psychometric characteristics of the BBI scales are shown in Table 4.

**Table 4:** Descriptive statistics of BBI scales (N = 361)

Descriptive statistics	EPE	DO	FBC	AD	ISER	EED	EOE	RPD	EDef	EAvo	DP	PPD	Alarm	Resistance	Exhaustion	BBI total score
Mean	8.92	7.07	7.22	6.50	16.29	10.78	7.57	13.38	9.08	10.73	6.14	5.20	29.76	47.98	31.15	108.97
SD	8.54	6.64	7.37	6.89	7.07	6.42	7.64	7.78	7.12	6.41	6.58	5.20	20.91	18.83	17.86	49.46
Kolmogorov-Smirnov Z	3.23	3.23	3.25	3.52	2.01	2.16	3.15	1.37	2.41	2.25	3.34	3.44	1.82	0.72	1.86	1.57
Asymp. Sig (2-tailed)	< 0.001	< 0.001	< 0.001	< 0.001	0.001	< 0.001	< 0.001	0.040	< 0.001	< 0.001	< 0.001	< 0.001	< 0.001	0.670	< 0.001	0.010
Cronbach’s α*	0.59	0.45	0.57	0.59	0.44	0.12	0.59	0.34	0.30	0.32	0.55	0.54	0.68	0.54	0.65	0.83

*M* mean, *SD* standard deviation, *Z* Z-score in Kolmogorov–Smirnov test, *Asymp. Sig. (2-tailed)* asymptotic significance (two-tailed) in Kolmogorov–Smirnov test, *, a measure of internal consistency, *EPE* experiencing psychotraumatic events, *DO* dissatisfaction with oneself, *FBC* feeling of being caged, *AD* anxiety and depression, *ISER* inadequate selective emotional response, *EED* emotional and ethical disorientation, *EOE* economy of emotions, *RPD* reduction of professional duties, *EDef* emotional deficit, *EAvo* emotional avoidance, *DP* depersonalization, *PPD* psychosomatic and psychovegetative disorders

As one of the main types of reliability, the internal consistency/homogeneity of the Boyko’s burnout inventory subscales ranged from 0.12 (emotional and ethical disorientation) to 0.59 (experiencing psychotraumatic events), thereby not reaching the value of 0.7 required by psychometric standards for any given scale. The scales corresponding to the phases of the syndrome development (alarm, resistance, and exhaustion) were closely related to each other: r = 0.55–0.62, p < 0.001 (Appendix 1).

The results of the factor analysis of the BBI scales are shown in Table 5. Confirmatory factor analysis of the questionnaire items was not carried out due to insufficient sample size (N = 361, while BBI includes 84 items).

**Table 5:** Factor matrix of BBI scales

BBI scales	Factors		
	1	2	3
Experiencing psychotraumatic events	0.81		
Dissatisfaction with oneself	0.30		0.72
Feeling of being caged			0.76
Anxiety and depression	0.73		0.31
Inadequate selective emotional response	0.38	0.66	
Emotional and ethical disorientation		0.47	0.42
Economy of emotions	0.60		
Reduction of professional duties	0.38	0.56	
Emotional deficit		0.50	0.38
Emotional avoidance		0.70	
Depersonalization	0.62	0.39	
Psychosomatic and psychovegetative disorders	0.80		
PVE, %	25.73	16.33	14.98

Factor loadings of 0.3 and higher are presented; PVE, proportion (%) of variance explained by the factor

### Correlation between MBI and BBI scales

**Fig. 1:**
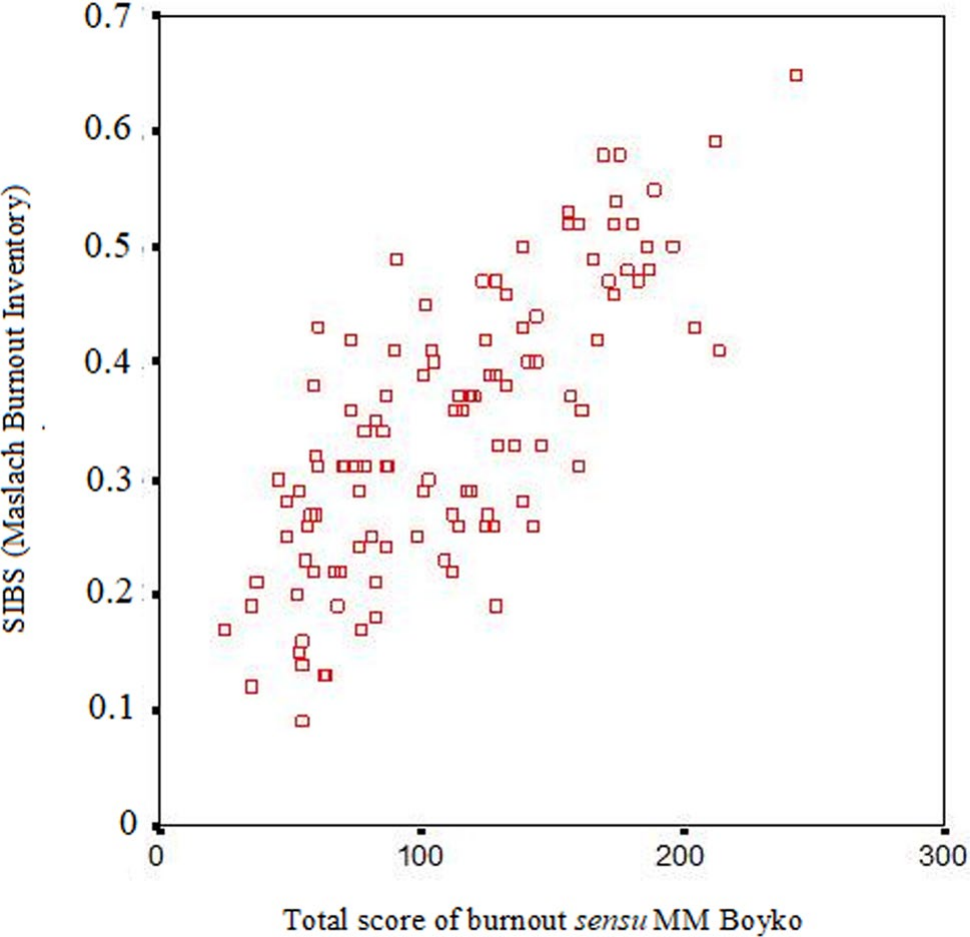
Scatterplot of occupational vs*.* emotional burnout

The correlation coefficients between the scales of two burnout inventories are presented in Table 6. All three MBI subscales correlated with the total burnout score according to the BBI (the sum of the scores across all 12 subscales). The maximum correlation coefficient was observed for the total scores of the two inventories (r = − 0.73, p < 0.001). A scatterplot for the total scores of the two questionnaires is shown in Fig. 1. Both inventories apparently assessed the same construct.

**Table 6:** Correlations of MBI and BBI scales

BBI subscales	MBI scales			
	EE	DP	PA	SIBS
Experiencing psychotraumatic events	− 0.53**	− 0.46**	0.03	− 0.45**
Dissatisfaction with oneself	− 0.50**	− 0.40**	0.38**	− 0.52**
Feeling of being caged	− 0.40**	− 0.29**	0.44**	− 0.45**
Anxiety and depression	− 0.45**	− 0.34**	0.14	− 0.40**
Inadequate selective emotional response	− 0.29**	− 0.46**	0.22*	− 0.43**
Emotional and ethical disorientation	− 0.06	− 0.09	0.28**	− 0.17
Economy of emotions	− 0.63**	− 0.42**	0.31**	− 0.57**
Reduction of professional duties	− 0.32**	− 0.32**	0.27**	− 0.38**
Emotional deficit	− 0.31**	− 0.43**	0.44**	− 0.49**
Emotional avoidance	− 0.03	− 0.29**	0.24*	− 0.25**
Depersonalization	− 0.56**	− 0.47**	0.18	− 0.51**
Psychosomatic and psychovegetative disorders	− 0.54**	− 0.37**	0.19*	− 0.46**
Alarm	− 0.67**	− 0.53**	0.32**	− 0.63**
Resistance	− 0.52**	− 0.51**	0.40**	− 0.61**
Exhaustion	− 0.49**	− 0.56**	0.40**	− 0.61**
Total score of burnout	− 0.68**	− 0.63**	0.44**	− 0.73**

*, p < 0.05; **, p < 0.01. *EE* emotional exhaustion, *DP* depersonalization, *PA* personal accomplishment, *SIBS* systemic index of burnout syndrome

Of the 64 correlations between the scales of the two inventories, only 7 (10.1%) were not statistically significant. The strongest correlations were established between the SIBS value and the total score of burnout on BBI scales, exhaustion score, alarm score, resistance score, and emotional burnout score.

Table 7 presents the results of the correlation analysis between the MBI scales and factors based on the BBI scales.

**Table 7:** Correlations between the scales of MBI and factors based on the BBI

MBI scales	BBI factors		
	Factor score 1	Factor score 2	Factor score 3
Emotional exhaustion	0.613**	0.181	0.438**
Depersonalization	0.490**	0.389**	0.180
Personal accomplishment	− 0.079	− 0.356**	− 0.373**

** p < 0.01 (two-tailed)

### Comparison of MBI and BBI by the Bland–Altman plot method

The Bland–Altman plot method was applied to compare MBI and BBI scores in two ways.

#### Method #1

**Fig. 2:**
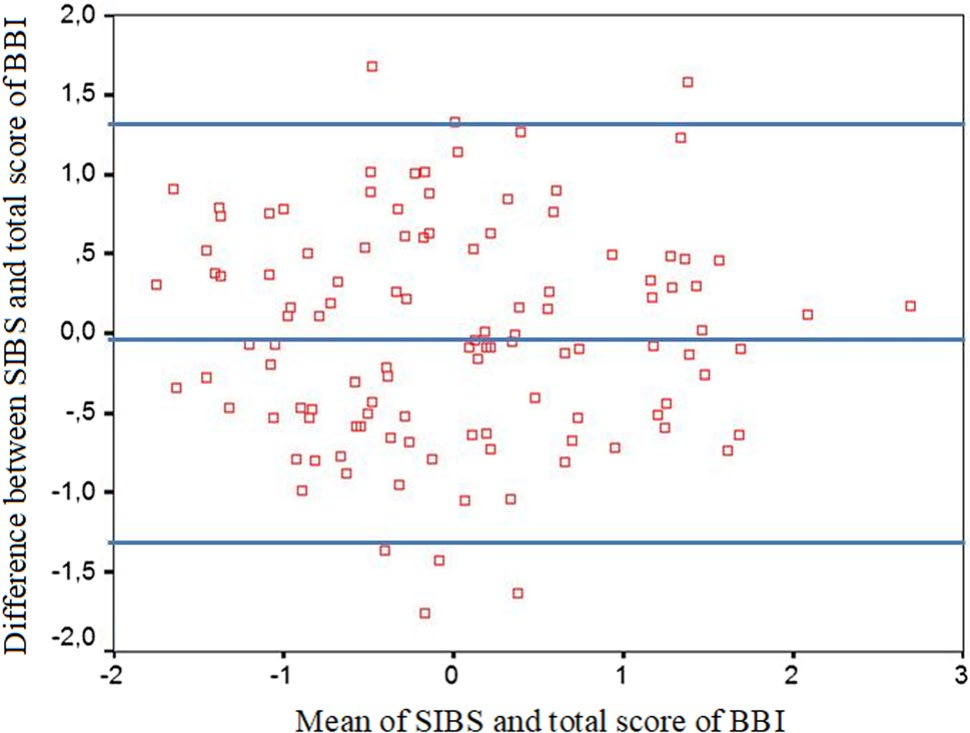
Plot of differences between MBI and BBI vs. the mean of two inventories (Method 1)

To construct a graph using the Bland–Altman method, we normalized the scales of both inventories. Without normalization, the use of the procedure for comparing two scales is impossible due to their different quantitative ranges: the SIBS value varies from 0 to 1, whereas the total score of burnout on the BBI scale ranges from 0 to 360. Normalization was carried out by subtracting the value of the sample arithmetic mean from the scale score; the resulting difference was divided by the value of the sample standard deviation. The means of the normalized scales were close to zero, the standard deviations were close to one. On the graph presented in Fig. 2, the *x-*axis represents the means of the normalized scores of the two indices, while the *y*-axis denotes the difference between the normalized values. The horizontal lines correspond to the arithmetic mean of the difference between the scores of the two scales (− 0.003), and to the upper and lower limits of the confidence interval (1.34 and − 1.35).

#### Method #2

To construct a graph using the Bland–Altman method, we unified the scales of both inventories, because without this procedure, it is impossible to compare them due to their different quantitative ranges: as mentioned above, the SIBS value varies from 0 to 1, whereas the total score of burnout on the BBI scale ranges from 0 to 360. Unification was carried out using the following formula:

$$x_{i}^{*} = \frac{{x_{i} - x_{\min } }}{{x_{\max } - x_{\min } }}.$$

**Fig. 3:**
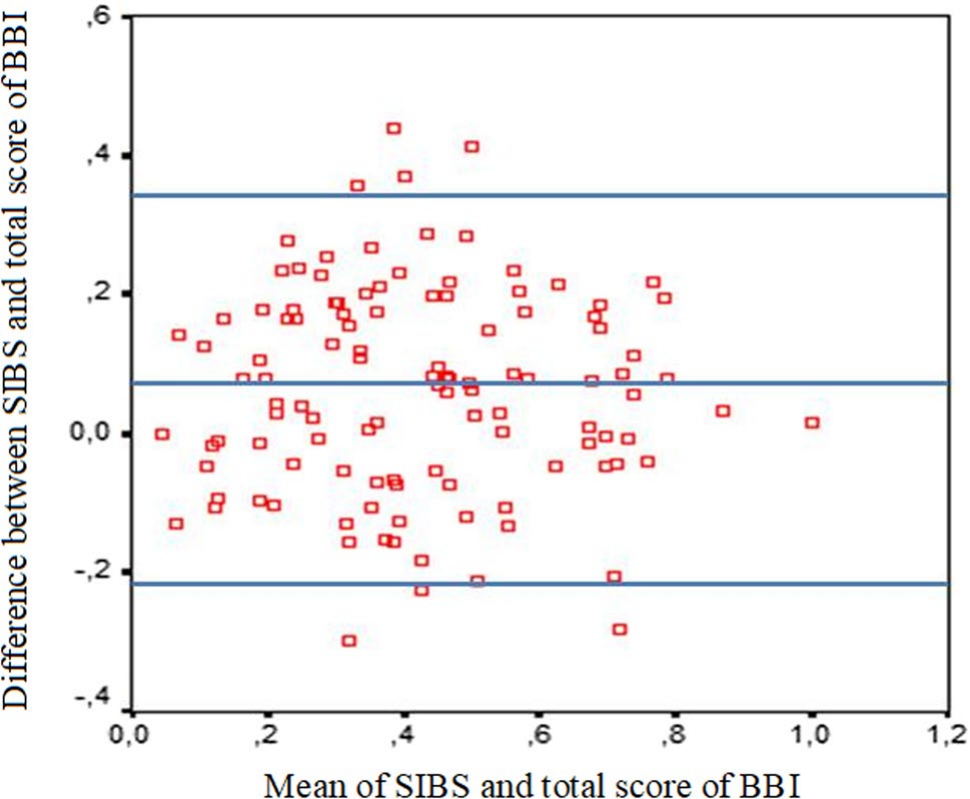
Plot of differences between MBI and BBI vs. the mean of two inventories (Method 2)

As a result of unification, the scores of both inventories were brought to the scale with a maximum score of 1 point. On the graph, the *x*-axis represents means of the unified scores of the two scales, while the *y*-axis represents the difference between the values of the unified scores (MBI–BBI). The horizontal lines correspond to the arithmetic mean of the difference between the unified scores of the two scales (0.06; standard deviation = 0.14), and to the upper and lower limits of the confidence interval (− 0.22 and 0.35) (Fig. 3). Therefore, the unified MBI score is slightly higher than the unified BBI score.

The limits of the confidence interval represent a more meaningful parameter for comparing unified scales than the differences between their arithmetic means. According to our data, 95% of the differences between the unified scales are within the range of values from − 0.22 to 0.35. Taking into account that the maximum difference between the ratings of unified scales for one particular research participant can be 1, and the minimum difference of the kind can be − 1 (negative one), the values of the confidence interval limits in our study imply that the ratings based on the two scales are consistent and lie within the same or adjacent evaluation categories in the vast majority of cases (e.g., well below average, below average, etc.).

### Cross-comparative analysis of MBI and BBI

To carry out the cross-comparative analysis for each scale, we determined the boundaries of the quartile intervals of the MBI and BBI scales. The scale scores for the subjects in the study sample were distributed among quartile intervals. E.g., 25% of the sample with the minimum scores on the scale were assigned to the first quartile. The results of the comparison are presented in Table 8. More than half of the subjects in the sample were in the same quartile, and about 40% were in neighboring quartiles. Thus, it can be argued that the results of assessing the severity of burnout by the two inventories were highly consistent with each other.

**Table 8:** Results of cross-comparative analysis of scales of occupational and emotional burnout questionnaires

	Number	%
Same cell	59	52.21
Adjacent cell	45	39.82
One cell over	9	7.96
Opposite cell	0	0.00

### Odds ratio

To calculate the OR, the scores on the four MBI scales and the total emotional burnout score on the BBI were converted into binary scales. On each scale, the subjects were divided into two categories: the first one included study subjects with scores falling within the boundaries of the third tertile (33 percent of the sample with high scale scores), whereas the second one comprised those with scores within the boundaries of the first and second tertiles.

To assess the correspondence between the measurement results of the MBI and BBI, the OR and the χ^2^ statistic were calculated for four pairs of binary scales. Consistency between BBI scores and four MBI scores (including the SIBS score) was analyzed (Table 9). For all obtained values of the OR criterion, the lower boundary of the confidence interval was above zero. The maximum OR value was obtained for the occupational burnout index. Therefore, in our sample, high values of total BBI score were directly associated with high values of MBI subscale scores and SIBS value.

**Table 9:** Correlation (associations) of BBI total score with MBI scale scores

MBI scales	OR	χ^2^	P	95% confidence interval	
				Lower boundary	Upper boundary
SIBS	20.53	43.58	< 0.001	7.53	55.99
Emotional exhaustion	15.51	36.74	< 0.001	5.83	41.27
Depersonalization	9.71	26.9	< 0.001	3.86	24.4
Personal accomplishment	0.16	12.02	< 0.001	0.05	0.49

### BBI score recalculation model for comparison with MBI results

We used regression analysis to develop a procedure for recalculating BBI scale scores into MBI scores. To build and compare regression models, we used solely SIBS, BBI alarm score, BBI resistance score, BBI exhaustion score, and 12 BBI scale scores. The assessment and choice of the model for constructing the recalculation formula was based on the value of the coefficient of determination (R^2^). Independent variables (BBI scales) with statistically insignificant values (p > 0.05) for predicting the SIBS were excluded from the models.

*Systemic index of burnout syndrome (SIBS).* To determine the SIBS, calculated by the formula proposed by the V.M. Bekhterev Institute (see *Methods* section), the regression models with the use of scores of three cumulative factors (alarm, resistance, exhaustion) were nearly as effective as the models using the total score of all 12 BBI subscales. The coefficients of determination (R^2^) were 0.585 (three factors) and 0.582 (sum of 12 BBI subscale scores). Therefore, for ease of use, we propose a recalculation formula based solely on the total score of BBI (sum of scores across 12 scales of BBI):

$${\text{SIBS}} = 0.{131} + 0.00{193} \times {\text{BBI total score}}.$$

The correlation coefficient between the SIBS obtained by MBI and the SIBS calculated by the above-mentioned formula was 0.763. The descriptive statistics calculated for these two scales were very close in magnitude: M = 0.345, σ = 0.091, X_min_ = 0.18, X_max_ = 0.60 (the original scale); M = 0.343, σ = 0.119, X_min_ = 0.09, X_max_ = 0.65 (new scale).

*Emotional exhaustion of MBI (EE).* The highest value of the coefficient of determination (0.638) was obtained for the regression model using the scores of all 12 BBI subscales:

$${\text{EE}} = 8.48 + 0.245{\text{EPE}} + 0.256{\text{DO}} + 0271{\text{FBC}} + 0.373{\text{EOE}} + 0.202{\text{DP}}.$$

where EE is emotional exhaustion, EPE is experiencing psychotraumatic events, DO is the dissatisfaction with oneself, FBC is a feeling of being caged, EOE is an economy of emotions, and DP is a depersonalization.

The correlation coefficient between the EE obtained via MBI and the EE calculated by the above formula was 0.799. The descriptive statistics were as follows: M = 18.708, σ = 8.578, X_min_ = 0, X_max_ = 43 (original scale); M = 18.698, σ = 6.848, X_min_ = 8.48, X_max_ = 39.37 (new scale).

*Depersonalization of MBI (DP).* The highest value of the coefficient of determination (0.437) was obtained for the regression model using the scores of 12 BBI subscales:

$${\text{DP}} = 0.185{\text{EPE}} + 0.129{\text{ DO}} + 0.93{\text{ ISER}} + 0.155{\text{ EDef}},$$

where EPE is experiencing psychotraumatic events, DO is the dissatisfaction with oneself, ISER is inadequate selective emotional response, and EDef is an emotional deficit.

The correlation coefficient between depersonalization calculated via the Maslach method and the values calculated by the formula of the regression equation was 0.661. The descriptive statistics were as follows: M = 8.21, σ = 4.96, X_min_ = 0, X_max_ = 21 (the original scale); M = 8.21, σ = 3.28, X_min_ = 1.38, X_max_ = 16.40 (the predicted values).

*Reduction of personal accomplishment of MBI (PA).* The highest value of the coefficient of determination (0.348) was obtained for the regression model using the scores of 12 BBI subscales:

$${\text{PA}} = {36}.{47}{-}0.{\text{185 DO}}{-}0.{\text{268 FBC}}{-}0.{\text{351EDef}} + 0.{19}0{\text{ DP}},$$

where DO is the dissatisfaction with oneself, FBC is a feeling of being caged, EDef is an emotional deficit, and DP is depersonalization.

The correlation coefficient between the reduction of personal accomplishment (PA) obtained for the study sample by the Maslach method and the reduction of PA calculated using the above formula was 0.59. The descriptive statistics were: M = 31.24, σ = 6.04, X_min_ = 18 X_max_ = 46 (the original scale); M = 31.24, σ = 3.56, X_min_ = 21.46, X_max_ = 37.04 (new scale of the regression model).

All regression models were statistically significant (F-test: p < 0.001). However, we obtained coefficients of determination (R^2^) exceeding 0.5 only for the first two models, which implied the acceptability of the model for the purpose of prediction. For the scales of DP and reduction of PA, these indicators of the model quality were 0.437 and 0.348, respectively.

## Discussion

The results of our study imply the possibility of comparing the scale scores of two most commonly used burnout inventories (BBI and MBI). Besides, our data highlighted some features (psychometric qualities) of the methods. Unfortunately, the search for publications describing the psychometric features of the Russian-language version of the MBI, as well as the BBI, did not yield results.

Our data demonstrated that both inventories (MBI and BBI) evaluate the same construct, which was confirmed by high statistical significance (89.9%) of correlations between the scales of the two questionnaires. It should be noted that an assessment of associations between individual subscales was beyond the scope of this study. Despite the fact that the inventories were originally developed on the basis of different theoretical models, the burnout symptoms covered by their content are interconnected, and the burnout syndrome severity is demonstrated as an amplified manifestation of its main components.

N.E. Vodopyanova and E.S. Starchenkova obtained similar results: the MBI scale of EE exhibited the highest number of associations with BBI scales [13]. Weaker correlations were obtained with the reduction of PA scale (the authors used the term ‘satisfaction with personal/professional accomplishments’). The authors explained these discrepancies by the lack of the possibility of assessing their own PAs in the BBI.

N.E. Vodopyanova and E.S. Starchenkova, along with other authors of similar studies, used correlation analysis (correlation coefficient) as a measure of similarity between MBI and BBI. Obtaining multiple statistically significant relationships between the scales of the two questionnaires is the basis for the conclusion about the conformity of the constructs assessed by their scores, as well as about the complementarity of information on the manifestations of the burnout syndrome provided by the subscales of the inventories (the so-called ‘completeness of symptoms’).

Despite the fact that the correlation between scale scores is a common method for assessing the degree of conformity of measurement results in psychological research, it is believed that the magnitude of the correlation coefficient does not provide the necessary completeness of information on the consistency of measurement results performed via different procedures [27]. In addition to correlation analysis, we employed the 1983 Bland–Altman plot method to compare the two burnout assessment procedures. We also used the cross-comparative analysis and OR statistic with 95% confidence interval. The results of statistical procedures suggested that there were no significant differences between the assessment of the burnout syndrome severity by the MBI and BBI methods in the study sample.

Analysis and comparison of factor matrices, for which items (MBI) and scales (both MBI and BBI) were used as initial material, made it possible to clarify the composition of the structural components of the burnout syndrome. As a result of the confirmatory factor analysis of the MBI items with the selection of three factors, we established that the first two factors of the model presented in Table 3 generally corresponded to the two components of the three-factor burnout model (viz., EE and PA). Items of the third scale (DP) were distributed across the first two factors.

The third factor included the following items with the maximum factor loadings: items 7 (*I can find the right solution in conflict situations arising when communicating with my colleagues*) and 17 (*I can easily create an atmosphere of goodwill and cooperation in my team*) of the PA scale, and item 6 of the EE scale (*After work, I feel like retiring from everyone and everything for a while*). Therefore, the third factor evaluates a certain set of communicative competencies, which, perhaps, do not depend on burnout as a phenomenon.

It is worth noting that in studies conducted in some European countries (e.g., France and Poland), the factor-based structures of MBI items matched the Maslach theoretical model: the items were distributed across three factors, which could be interpreted as EE, reduction of PA and DP (cynicism) [28]. According to our study, the factor-based structures of MBI items in Russia differ from those in European countries, which can be explained by its incomplete adaptation.

As a result of the analysis of associations between the MBI scales (Table 1), we obtained statistically significant correlation coefficients between all three scales, including a negative and rather strong association of EE with a reduction in PA (r = − 0.37, p < 0.001). In other well-known studies that have used the MBI, these two scales did not correlate with each other. The relationship between EE and reduction of PA in our study can be explained by specific features of our sample (viz., age and gender composition); i.e., the most active and productive age stage in men is likely to be associated with professional success and wellbeing to a greater extent.

Analysis of the factor-based structure of the BBI scales (Table 5) leads to the conclusion that the first and second factors of the BBI with the proportions of variance explained by the factor (PVE) equal to 25.73 and 16.33, respectively, of the obtained three-factor model, apparently, can be linked to the conventional factors sensu C. Maslach: EE and DP (emotional detachment). The third factor (PVE = 14.98) includes two BBI scales with maximum weights related to the alarm phase sensu Boyko: dissatisfaction with oneself and feeling of being caged. The weights of other scales in the third factor are insignificant. Interpreting the third factor as a loss of self-confidence (reduction in self-efficacy), we can notice its similarity with the descriptors of the MBI reduction of PA scale: a decrease in the sense of competence and productivity arising from the inability to cope with the requirements at work. The similarity of the third factor with the reduction of PA scale is also specified by the fact that the PA scale sensu Maslach most strongly correlates with this third factor (r = − 0.373, p < 0.01) (Table 5). For C. Maslach, reduction of PA as a measurement of professional burnout is self-evaluative—i.e., it affects a person’s assessment of own professional achievements. It can be assumed that the resulting factor specifically reflects the emotional response to the situation as a whole, where professional success is only one of the components of the current status quo. Hence, despite the differences in theoretical approaches to burnout models, statistical analysis reveals the similarity of the factor-based structures of the studied questionnaires. Accordingly, the factor-based structure of the BBI scales replicates the C. Maslach model in its main characteristics.

Unfortunately, there are no data on the psychometric properties of the original Russian-language version of the BBI, but there is experience in adapting and validating the BBI in Bulgaria [19]. For the Bulgarian-language version of BBI, statistically significant moderate to strong correlation coefficients (P < 0.01) were obtained for all subscales with MBI scales, which was construed by the researchers as high validity. According to the authors of the Bulgarian version of BBI scales, they exhibited high reliability: e.g., for the stress phase scales and the final scale of homogeneity assessment (Cronbach’s alpha), indicators of reliability ranged from 0.797 to 0.944. In our study, somewhat lower albeit acceptable values of the Cronbach’s alpha criterion were obtained for the phase scales and the cumulative scale of burnout: from 0.54 (resistance score) to 0.84 (total score). The established correlations between the scales of the two inventories can also be interpreted as confirmation of the BBI validity.

The performed statistical analysis resulted in obtaining the regression models for recalculating BBI scale scores into MBI scale scores. For two MBI scales, the quality of the models was sufficiently high; for the other two, it was classified as satisfactory. The lowest coefficient of determination was obtained for the regression model of the reduction of PA, which indicated the areas of discrepancy between the phenomenology covered by the two inventories.

The problem of burnout, the relevance of which has not yet diminished, requires the availability of burnout assessment tools that have the necessary psychometric qualities. The data obtained in our study revealed a connection between the results of the V.V. Boyko’s questionnaire for studying burnout (common in Russia and some foreign countries) with C. Maslach’s questionnaire, which serves as the gold standard for researchers and practitioners in assessing burnout. Considering the popularity of the Boyko questionnaire in studies of the burnout phenomenon and taking into account the large number of scales included in it, the identified connections between the scales of the two questionnaires allow us to better understand the symptoms of burnout. Ultimately, this will contribute to a more accurate identification of individual symptoms of burnout syndrome, their combinations and diagnosis, which will properly affect the effectiveness of preventive measures and measures to provide psychological assistance.

Since this study was carried out on a sample that included only men, further research should be aimed at identifying associations between the MBI and BBI scales in a female sample. Furthermore, we think that our data indicate the need to conduct a psychometric study with subsequent revision and modification of the Russian version of the BBI questionnaire. With the advent of new versions of questionnaires based on C. Maslach’s model for studying burnout, as well as burnout questionnaires based on more recent models of burnout (e.g., Burnout Assessment Tool (BAT) [29]), further comparative studies seem relevant. Such studies should be aimed at clarifying the content of psychological constructs and evaluating their psychometric qualities.

## Conclusion

Based on the results of our study, we advocate that both MBI and BBI yield largely consistent results. Hence, we suggest that the data sets obtained in studies using these questionnaires can be compared with each other after the required recalculation is performed. Proposed linear conversion factors for BBI to MBI are presented in the main text of the article.

### Advantages and limitations of the study

The study was carried out on a homogeneous group of individuals (in terms of gender composition, age composition, and living conditions: men, 41–44 years old, residents of Moscow.

However, our research had some shortcomings: its major limitation was an absence of women among study subjects, especially considering the results of some studies on the gender-based specificity of the burnout syndrome [19, 27].

## Data Availability

The data that support the findings of this study are not openly available and are available from the corresponding author upon reasonable request.

## Appendix 1. Correlations between BBI scales (N = 360)

**Table:**

BBI Scales		DO	FBC	AD	ISER	EED	EE	RPD	EDef	EAvo	DP	PPD	Alarm	Resistance	Exhaustion	Burnout
EPE	r	0.22	0.15	0.49	0.24	− 0.06	0.39	0.25	0.23	0.02	0.44	0.56	0.69	0.33	0.43	0.58
	p	0.00	0.00	0.00	0.00	0.28	0.00	0.00	0.00	0.66	0.00	0.00	0.00	0.00	0.00	0.00
DO	r		0.49	0.35	0.21	0.21	0.35	0.33	0.41	0.24	0.43	0.29	0.70	0.43	0.49	0.64
	p		0.00	0.00	0.00	0.00	0.00	0.00	0.00	0.00	0.00	0.00	0.00	0.00	0.00	0.00
FBC	r			0.38	0.20	0.24	0.25	0.35	0.28	0.28	0.36	0.27	0.69	0.40	0.42	0.60
	p			0.00	0.00	0.00	0.00	0.00	0.00	0.00	0.00	0.00	0.00	0.00	0.00	0.00
AD	r				0.27	0.08	0.39	0.35	0.24	0.05	0.48	0.55	0.77	0.44	0.45	0.66
	p				0.00	0.13	0.00	0.00	0.00	0.38	0.00	0.00	0.00	0.00	0.00	0.00
ISER	r					0.16	0.26	0.37	0.25	0.20	0.36	0.28	0.32	0.68	0.39	0.54
	p					0.00	0.00	0.00	0.00	0.00	0.00	0.00	0.00	0.00	0.00	0.00
EED	r						0.07	0.17	0.29	0.26	0.19	0.07	0.16	0.50	0.30	0.36
	p						0.19	0.00	0.00	0.00	0.00	0.17	0.00	0.00	0.00	0.00
EOE	r							0.33	0.34	0.12	0.40	0.41	0.49	0.66	0.45	0.62
	p							0.00	0.00	0.02	0.00	0.00	0.00	0.00	0.00	0.00
RPD	r								0.33	0.31	0.41	0.31	0.45	0.74	0.49	0.65
	p								0.00	0.00	0.00	0.00	0.00	0.00	0.00	0.00
EDef	r									0.32	0.39	0.28	0.40	0.46	0.74	0.61
	p									0.00	0.00	0.00	0.00	0.00	0.00	0.00
EAvo	r										0.31	0.06	0.20	0.34	0.62	0.44
	p										0.00	0.24	0.00	0.00	0.00	0.00
DP	r											0.57	0.60	0.53	0.80	0.75
	p											0.00	0.00	0.00	0.00	0.00
PPD	r												0.59	0.43	0.64	0.64
	p												0.00	0.00	0.00	0.00
Alarm	r													0.56	0.63	0.86
	p													0.00	0.00	0.00
Resistance	r														0.63	0.84
	p														0.00	0.00
Exhaustion	r															0.87
	p															0.00

*EPE* experiencing psychotraumatic events, *DO* dissatisfaction with oneself, *FBC* feeling of being caged, *AD* anxiety and depression, *ISER* inadequate selective emotional response, *EED* emotional and ethical disorientation, *EOE* economy of emotions, *RPD* reduction of professional duties, *EDef* emotional deficit, *EAvo* emotional avoidance, *DP* depersonalization, *PPD* psychosomatic and psychovegetative disorders
